# Analysis of suitable areas for *Phlebotomus chinensis* as vector of visceral leishmaniasis—China

**DOI:** 10.3389/fcimb.2026.1846723

**Published:** 2026-05-20

**Authors:** Zhongqiu Li, Ben Ma, Zixin Wei, Zhengbin Zhou, Limin Yang, Junhu Chen, Yi Zhang, Shizhu Li

**Affiliations:** 1 National Institute of Parasitic Diseases, Chinese Center for Disease Control and Prevention (Chinese Center for Tropical Diseases Research), National Health Commission (NHC) Key Laboratory of Parasite and Vector Biology, World Health Organization (WHO) Collaborating Center for Tropical Diseases, National Center for International Research on Tropical Diseases, National Key Laboratory of Intelligent Tracking and Forecasting for Infectious Diseases, Shanghai, China; 2 Department of Disinfection and Vector Control, Dongying Center for Disease Control and Prevention, Dongying, China; 3 Institute of Infectious Disease Prevention and Control, Shanghai Center for Disease Control and Prevention, Shanghai, China; 4 School of Global Health, Chinese Center for Tropical Diseases Research, Shanghai Jiao Tong University School of Medicine, Shanghai, China

**Keywords:** jackknife, leishmania, MaxEnt model, suitable areas, visceral leishmaniasis

## Abstract

**Objective:**

*Phlebotomus chinensis* (*P. chinensis*) serves as the primary vector of visceral leishmaniasis, a persistent public health threat, and its suitable distribution range and expansion trends remain unclear.

**Methods:**

This study integrated two sets of baseline data, with 24,281 specimens collected from 45 counties across six provinces through field surveys and extra records from 475 distribution sites collated via literature retrieval. Climate data with 19 bioclimatic variables and geographic data including elevation, slope, aspect and vegetation coverage were obtained from open-access online databases (data retrieval cutoff: 2023). The Maximum Entropy (MaxEnt) model was applied to simulate habitat suitability. The area under the receiver operating characteristic curve was used to evaluate model performance, and the Jackknife test was conducted to determine the relative importance of each environmental factor.

**Results:**

The MaxEnt model produced an outstanding AUC value of 0.917 ± 0.007, demonstrating robust predictive performance. Vegetation coverage emerged as the primary environmental driver, followed by the minimum temperature of the coldest month (bio_6), mean temperature of the driest quarter (bio_9), and annual precipitation (bio_12). The contemporary suitable habitat for *P. chinensis* on the Chinese mainland spans 2,295,360 km², constituting 23.91% of the total land area. Core suitable habitats concentrate in central Shanxi, northern and western Henan, southern Shaanxi and Gansu, southwestern Hebei, and northern Sichuan. Under the RCP4.5 scenario, the suitable distribution range will expand to 2,834,001 km² (29.52%) by 2050 and 2,934,104 km² (30.56%) by 2070. The centroid of current suitable habitats is situated in Dagui Town, Pingli County, Ankang City, Shaanxi Province (109.137665°E, 32.438831°N), with a northwestward shift of 112.37 km by 2050 and 100.15 km by 2070.

**Conclusions:**

This study potentially delineates the suitable ecological niche of *P. chinensis* across the Chinese mainland, identifies key environmental drivers, and provides scientific references for regional risk assessment and targeted prevention measures.

## 1 Introduction

Visceral leishmaniasis (VL), also known as Kala-azar, is a zoonotic disease caused by *leishmania* and transmitted by infected sandflies (Ghodrati et al., 2022). It is widely distributed in tropical and subtropical areas and some temperate areas (Kato et al., 2019). Among the sandfly, the distribution of *P. chinensis* is extensive, and it is also the main reason cause of VL in China. VL is a systemic disease with many clinical manifestations, including fever, hepatosplenomegaly, anemia, and liver dysfunction (Hassan et al., 2023).The World Health Organization also considers VL as neglected tropical disease, and it poses a serious public health risk, with an estimated 700,000 to 1 million new cases recorded worldwide each year (Maxfield and Crane, 2024). Among all parasitic diseases, VL is the second most common cause of death after malaria (Kelleci et al., 2023).

In the early 1950s, VL was widespread in more than 600 counties in 16 provincial-level administrative divisions (PLADs), all of which are north of the Yangtze River, with hundreds of thousands of cases recorded every year (Wang et al., 2012). After the large-scale control and elimination of sandflies, the disease was basically under control in most of the formerly endemic areas of the country in 1960s. The distribution of *P. chinensis* has been confirmed to be influenced by the environment (Guernaoui et al., 2006).Moreover, as a result of global warming, the prevalence and prevalence of VL have increased across the world (Mohebali, 2013). During the 21st century, with the development of society and the changing environment, VL has revived in historical endemic areas. Its incidence rate has increased in recent years (Guan et al., 2021). In 2022, 239 cases of VL were reported in 104 counties in 11 provinces with scattered distribution, mainly being found in Hebei, Shaanxi, Henan and Shaanxi provinces, accounting for 79.3% of cases in China (Li et al., 2023). P*. chinensis* are now widely distributed in China, being recorded in 21 provinces (autonomous regions and municipalities) and about 358 counties (Tang et al., 2012).Therefore, understanding the distribution of sandflies is of great significance for the prevention and control of VL in China.

The maximum entropy model (MaxEnt model) was used to evaluate the potential distribution of sandflies based on environmental resources and geographical location (Carvalho et al., 2015). According to the published literature, the environmental factors that affect the distribution of VL are daily precipitation and precipitation during the driest month, the maximum temperature of the warmest quarter of the year and the daily average temperature determined by the MaxEnt model (Gao and Cao, 2019). Khamesipour et al. found that the distributions of *phlebotomus papatasi* and *phlebotomus sergenti* were influenced by slope factors in the MaxEnt model (Khamesipour et al., 2020). It has also been reported that the sandfly has a wide spread geographic distribution in Morocco. In addition, the greatest influence factors on that model’s development are annual precipitation and precipitation in the driest quarter of the year (Daoudi et al., 2020). *Sergentomyia minuta* was also found to have a wide geographical distribution in Morocco (Daoudi et al., 2020). particularly in northern and central Morocco. Precipitation seasonality, precipitation in the driest month and annual precipitation were the most significant factors. These examples illustrate that the MaxEnt model could not only predict the potential distribution of the *P. chinensis* but also found important ecological variables.

This study conducted a field survey of the distribution of the *P. chinensis* in 6 PLADs of China conducted from May to September 2023. The *P. chinensis* were screened to predict the potential distribution, which combined with the data in the literature on the distribution of the *P. chinensis*. MaxEnt model were used to study the distribution trend and influencing factors of the *P. chinensis* in China. Comprehending and mastering the distribution trend of *P. chinensis* is of great significance to control the VL in China.

## 2 Materials and methods

### 2.1 Distribution data of *P. chinensis*

We used two methods to obtain distribution information of sandflies:

1. Literature search: Keywords including “Sandfly”, “*P. chinensis*”, and “China” were used to search Chinese databases (CNKI, VIP, Wanfang) and English-language databases (Web of Science, PubMed) for relevant literature published from 1950 to 2023. After removing duplicates and excluding studies lacking geographic information or with unclear geographic information, 277 eligible studies (145 English, 132 Chinese) were retained, yielding 475 unique distribution sites. For papers without clear latitude and longitude, we used Google Earth (http://ditu.google.cn/) to geolocate the place names.

**Figure 1:**
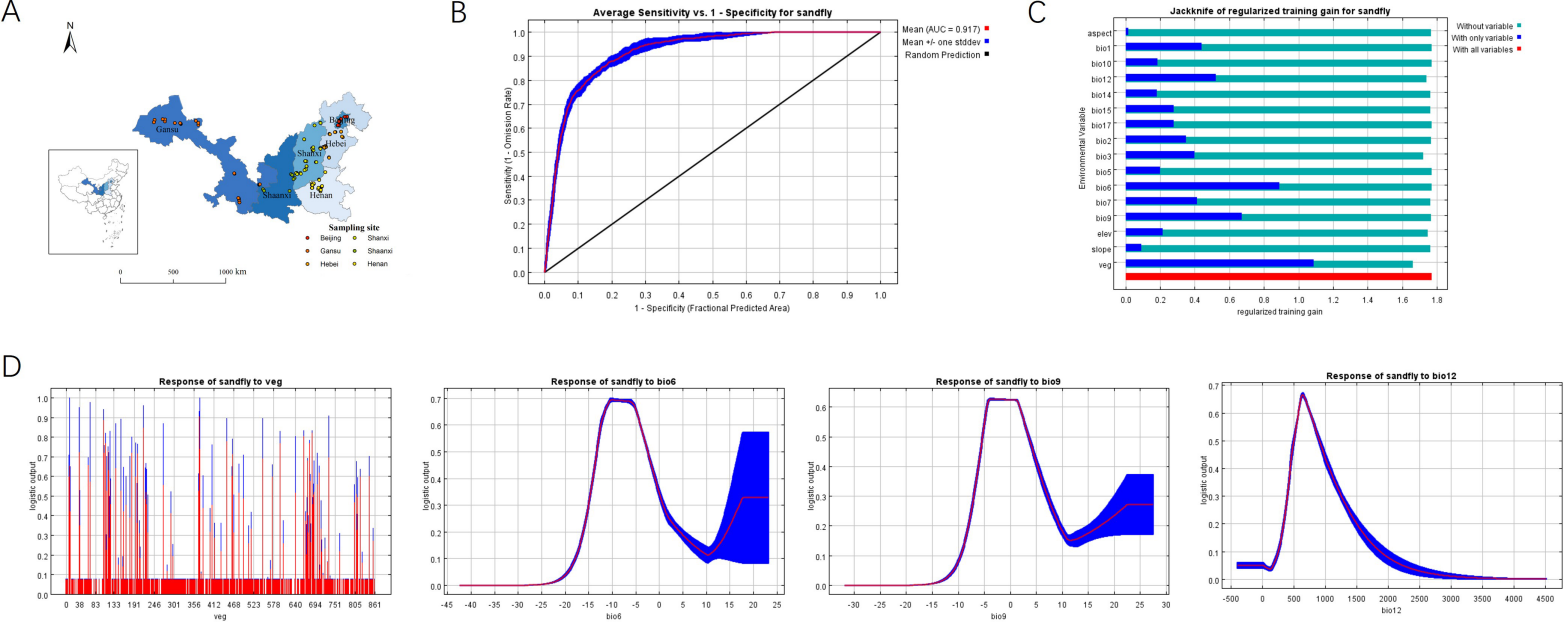
Distribution of sampling sites, receiver operating characteristic (ROC) curve with area under the curve (AUC), and relative contributions of environmental variables. **(A)** Distribution of sampling points in endemic and potential risk areas of VL in China. All sampling locations from field surveys (45 counties, 6 provinces) and literature-derived points are marked on the map. **(B)** ROC curve of *P. chinensis* MaxEnt maximum entropy model. **(C)** Importance analysis of environmental factors based on Jackknife method. **(D)** Response curves of dominant climate and environmental factors for *P. chinensis*.

Field investigation: The field investigation strictly followed the “National Visceral Leishmaniasis Surveillance Program”. We collected sandflies at each sampling site between 6 p.m. on the first day and 7 a.m. the next day using sandfly lamps in 2023. Sandflies collected from traps were taken back to the laboratory, stored at -80 °C after morphological identification, and only *P. chinensis* were counted. Notably, we did not calculate *P. chinensis* density for each survey point, county, city, or PLAD. The rationale for this design is that our primary objective was to determine presence/absence and relative abundance for habitat suitability modeling, rather than to estimate absolute population density. Presence-only models like MaxEnt are robust to the absence of density data, as they rely on occurrence records rather than abundance measures. Therefore, this approach does not compromise the accuracy of subsequent habitat suitability predictions. The sampling locations are marked in **Figure 1A**.

Regarding the representativeness of the field survey: Although *P. chinensis* is distributed across 21 provinces in China, our survey focused on 6 provinces (Shaanxi, Shanxi, Gansu, Hebei, Beijing, Henan) that represent the current VL-endemic regions and potential risk areas identified by the National Surveillance Program. These provinces have historically reported the highest VL case numbers and *P. chinensis* activities. Combined with the literature-derived distribution points covering additional provinces, our dataset captures the ecological range of *P. chinensis* across different climatic zones, ensuring nationwide representativeness for MaxEnt modeling.

### 2.2 Climatic and environmental data

Climate data were obtained from the World Claim Global Climate data website (www.worldclim.org). The World Claim Global Climate Data website is an open-source database of high-resolution global weather data, including current and future bioclimatic data. All the data are available for download. There are four spatial resolutions on the World Claim Global Climate Data website: 10Min (20Km), 5Min (10Km), 2.5Min (5Km) and 30Sec (1Km). This study selected are solution of 2.5Min (5Km) and included 19 bioclimatic factors, such as annual mean temperature, annual mean precipitation and isotherms. These data contained annual trends and seasonal features typical of the climate (Campos-Soldini, 2022). A Representative Concentration Pathway (RCP) is “a consistent forecast of time trends in radiation-active gas, particulate matter emissions, and concentration changes, a collection of widely covered anthropogenic effects on climate”. According to the radiation intensity level of 2.6~8.5w/m (Kato et al., 2019), RCPs are divided into four paths: the lowest forced horizontal path (RCP 2.6); two intermediate stable paths (RCP4.5/RCP 6.0), with RCP4.5 having higher priority than RCP6; and the most widely used high baseline emission path (RCP8.5) (Enari and Sakamaki-Enari, 2013).

This study selected the RCP4.5 scenario for future projections because it represents a moderate emissions pathway that aligns with China’s current commitments to carbon neutrality and climate mitigation policies. RCP4.5 is widely used in Chinese ecological niche modeling studies as a balanced scenario between extreme (RCP8.5) and optimistic (RCP2.6) projections, and it is consistent with China’s projected climate warming trends under moderate mitigation efforts^19^. The new versions of RCPs consider in more detail the response strategies to climate change and the impact on future greenhouse gas emissions and scientifically build models to predict future climate change (Hu et al., 2015). Elevation, slope, and aspect were derived from a digital elevation model (DEM, http://www.gscloud.cn/) and vegetation information (https://www.resdc.cn/) with a spatial resolution of 25 m. The climate and environmental factors used in this study are shown in **Table 1**.

**Table 1:** Environmental variables used in MaxEnt model.

Variable name	Definition	Variable name	Definition
bio_1	Annual mean temperature	bio_11	Mean temperature of coldest quarter
bio_2	Mean monthly temperature range	bio_12	Annual precipitation
bio_3	Isothermality	bio_13	Precipitation of wettest month
bio_4	Temperature seasonality	bio_14	Precipitation of driest month
bio_5	Max temperature of warmest month	bio_15	Precipitation seasonality
bio_6	Min temperature the coldest month	bio_16	Precipitation of wettest quarter
bio_7	Temperature annual range	bio_17	Precipitation of driest quarter
bio_8	Mean temperature of wettest quarter	bio_18	Precipitation of warmest quarter
bio_9	Mean temperature of driest quarter	bio_19	Precipitation of wettest month
bio_10 Veg	Mean temperature of warmest quarter vegetation	Elev Slop Aspe	elevation slope aspect

### 2.3 The acquisition of administrative zoning maps in China

We downloaded the 1:4,000,000 administrative district map of China (Basemap Approval Number: GS(2024)0650) from the National Basic Geographic Information System (http://www.ngcc.cn/). The map includes provincial boundaries; coastlines; the Hong Kong, Macao and Taiwan regions; and the South China Sea Islands, which were used as the base map for the prediction of the distribution of suitable areas.

### 2.4 Preprocessing for species distribution

To avoid over-fitting the prediction results due to the excessive concentration of distribution data, it is necessary to preprocess species distribution data via the buffer data screening method. The specific operation involved up loading the distribution points to ArcGIS 10.7 and displaying the x axis and y axis coordinates of the distribution points; then, we used the projection and transformation options in the data management tool to project the distribution points and defined the projection coordinates with WGS1984. Then, a buffer was set for the projected distribution points, and the radius of the buffer was independently set according to the size of the research area.

To avoid over-fitting the prediction results due to excessive concentration of distribution data, it is necessary to preprocess species distribution data via a buffer data screening method. The specific operation involved uploading the distribution points to ArcGIS 10.7 and displaying the x-axis and y-axis coordinates; then, we used the projection and transformation options in the data management tool to project the distribution points and defined the projection coordinates with WGS1984. Then, a buffer was set for the projected distribution points. In this study, a buffer zone with a radius of 5 km was set. This radius was chosen for two reasons: (1) it matches the spatial resolution of the climatic and environmental data (2.5Min ≈ 5 km at the equator), ensuring consistency between species occurrence data and environmental layers; (2) it reduces sampling bias from spatially clustered occurrence points while maintaining ecological relevance, as 5 km approximates the typical dispersal range of P. chinensis and avoids over-filtering valid distribution records. After this step, a circle with equal radius appeared around each projection point. The intersection option for overlay analysis was selected in the analysis tool, overlay analysis was performed on the buffer, and we found the intersection. Only one distribution point was retained in the intersecting buffer.

### 2.5 Screening of climate and environmental factors

To avoid autocorrelation between climate variables affecting the accuracy of prediction results, the variables were screened. Climate factors were screened in three steps:

First, the contribution degree of each climate factor to distribution prediction was estimated. Without considering collinearity, all original climate factor data were imported into the MaxEnt model to check the contribution rate of each environmental factor, which was used as the basis for subsequent screening. Second, the correlation between various factors was analyzed. Environmental data and species distribution data were uploaded to ArcGIS 10.7, and climate and environmental factors were resampled.

Third, the correlation between sampling results was analyzed using SPSS software (The University of Auckland, New Zealand). The Pearson correlation coefficient was calculated, and a correlation coefficient of |r| ≥ 0.9 was defined as high correlation. According to the contribution degree of climate factors predicted in the first step, the two highly correlated variables were screened, and the one with the higher contribution rate was retained. The following variables were excluded due to high correlation: bio_7 (temperature annual range, correlated with bio_4 and bio_5), bio_8 (mean temperature of wettest quarter, correlated with bio_1 and bio_10), bio_13 (precipitation of wettest month, correlated with bio_12), bio_14 (precipitation of driest month, correlated with bio_17), bio_15 (precipitation seasonality, correlated with bio_3), bio_16 (precipitation of wettest quarter, correlated with bio_13), bio_18 (precipitation of warmest quarter, correlated with bio_19), and bio_19 (precipitation of coldest quarter, correlated with bio_14). Ultimately, 16 environmental factors were included in model construction.

### 2.6 Prediction of suitable areas for sandflies under current and future climate model

The current species distribution data and climate factors were imported into the Sample File and Environment columns of the MaxEnt model software, respectively. We set the Logistic in Output as the output format, set the model parameters, randomly selected 75% of the distribution points as the training set, and used the remaining 25% as the test set to verify the model. We selected the average of the results of 10 repeated operations to cross-validate and effectively reduce the sampling error. We uploaded the species distribution data file to the Test sample, and the remaining options were used as default. The MaxEnt model was then completed.

The MaxEnt model established a receiver operating characteristic curve (ROC curve) between environmental factors and the distribution probability, and the size of the area under the ROC curve (AUC value) was used as a measure of the model’s prediction accuracy. The AUC value was proportional to the accuracy of the simulation results. Generally, when the AUC value was0.5~0.6, the prediction results failed, while 0.6~0.7 was poor, 0.7~0.8 was average, 0.8~0.9 was good, and 0.9~1.0 was excellent. The closer the model was to model 1, the more accurate the predicted results, and the stronger the correlation between species distribution and climate variables (Carmona-Castro et al., 2018). The jackknife method was used to analyze the importance of climate factors for species distribution in the MaxEnt model. The advantage of the jackknife method is that it can reduce the prediction bias and give approximate confidence intervals for multiple parameters (Larson et al., 2010). In this study, future climate data (2050; 2070) under the RCP4.5 path were used to predict the distribution of the future habitat of sandflies. The center of mass transfer and suitable areas were analyzed using ArcGIS 10.7 and SDM Toolbox.

### 2.7 Visualization of prediction results

The prediction results of the MaxEnt model were visualized using ArcGIS 10.7, and the predicted levels of the suitable areas were divided into four levels: highly suitable, medium suitable, low suitability, and unsuitable. The distribution map of the suitable areas was drawn by using the 1:4,000,000 administrative zoning maps as the base map.

## 3 Results

### 3.1 Investigation of *P. chinensis* in endemic and potential VL risk areas in China

According to the distribution of endemic and potential VL risk areas in China, a random survey was carried out on 45 counties in 6 provinces in China (**Figure 1A**). A total of 24,281 *P. chinensis* were collected in this survey; among them, the most samples were collected in Linfen City (4774, 19.66%), Jiexiu City (4374, 17.90%), and Wuxiang town (4002, 16.48%) in Shanxi Province, respectively (**Table 2**). At several sampling sites in Gansu Province, including Jinta, Guazhou, and Dunhuang, no *P. chinensis* were captured. These sites are characterized by extremely arid conditions with annual precipitation below 100 mm and sparse vegetation coverage, which fall outside the optimal ranges identified for *P. chinensis* survival.

**Table 2:** Sampling of surveillance counties in endemic areas and potential risk areas of VL in China.

Province	City/County	The number of *Phlebotomus chinensis*	Percentages(%)
Shaanxi	Yan’an	75	0.31
	Hancheng	400	1.65
	Huazhou district	81	0.33
	Qianyang	538	2.22
Shanxi	Hongdong	21	0.09
	Huozhou	19	0.08
	Ji	2267	9.34
	Jiexiu	4374	18.01
	Wuzhai	44	0.18
	Yanggao	12	0.05
	Yangqu	112	0.46
	Yingze	85	0.35
	Yungang	79	0.33
	Yangquan	642	2.64
	Linfen	4774	19.66
	Wuxiang	4002	16.48
Gansu	Jinta	0	0.00
	Guazhou	0	0.00
	Dunhuang	0	0.00
	Yumen	5	0.02
	Gaolan	65	0.27
	Huating	522	2.15
	Tanchang	739	3.04
Hebei	Jingxing	629	2.59
	Jingxing mining area	54	0.22
	Xingtai	5	0.02
	Gaobeidian	0	0.00
	Layuan	0	0.00
	Renqiu	0	0.00
	Yi	19	0.08
Beijing	Changping district	17	0.07
	Fangshan district	0	0.00
	Miyun district	0	0.00
	Haidian district	10	0.04
Henan	Bo’ai	661	2.72
	Jiyuan	548	2.26
	Jiashi	410	1.69
	Yiyang	513	2.11
	Yuzhou	380	1.57
	Linzhou	517	2.13
	Xingyang	510	2.10
	Gongyi	83	0.34
	Dengfeng	569	2.34
	Mengjin district	500	2.06
Total	/	24281	100

### 3.2 Data of *P. chinensis* and accuracy verification of MaxEnt model

In the literature, there were 145 English-language studies and 132 Chinese studies that met the inclusion criteria. A total of 475 distribution sites for sandflies were obtained from these studies. Based on the results of sampling points, there were 790 distribution sites in total. After screening all distribution sites with 5Km buffer (the same resolution as climate and environmental factors), 366 distribution sites were obtained for the construction of the maximum entropy model. To verify the accuracy of the prediction model, if the average AUC equaled 0.917 ± 0.007, including the training set and the test set, the prediction effect of the model was accurate (**Figure 1B**).

### 3.3 Effects of climate and environmental factors on the distribution of current *P. chinensis* habitats

Among the factors influencing the habitat suitability distribution of *P. chinensis*, vegetation coverage (veg) has the greatest impact. it indicates that vegetation types such as temperate semi-shrubland, short semi-shrubland desert, temperate mountain coniferous forest, and temperate deciduous broad-leaved forest are suitable for the reproduction of *P. chinensis*. These vegetation types provide specific microhabitats: temperate deciduous broad-leaved forests offer leaf litter and soil crevices for larval development and resting sites for adults; temperate semi-shrublands and short semi-shrubland deserts provide shelter from extreme temperatures and serve as breeding grounds due to organic matter accumulation. Following vegetation coverage is the minimum temperature of the coldest month (bio_6), the mean temperature of the driest quarter (bio_9), and annual precipitation (bio_12) (**Figure 1C**). The response curves between dominant environmental factors and the probability of P. chinensis distribution all exhibit a bell-shaped pattern, meaning that as environmental conditions change, the probability of *P. chinensis* distribution initially increases and then decreases (**Figure 1D**). Areas where the minimum temperature of the coldest month ranges from -14.10 °C to -3.21 °C, the mean temperature of the driest quarter ranges from -5.37 °C to 3.80 °C, and annual precipitation falls between 500 mm and 862 mm are suitable for *P. chinensis* survival.

### 3.4 Prediction results of suitable areas for *P. chinensis*, under the current climate model

**Figure 2:**
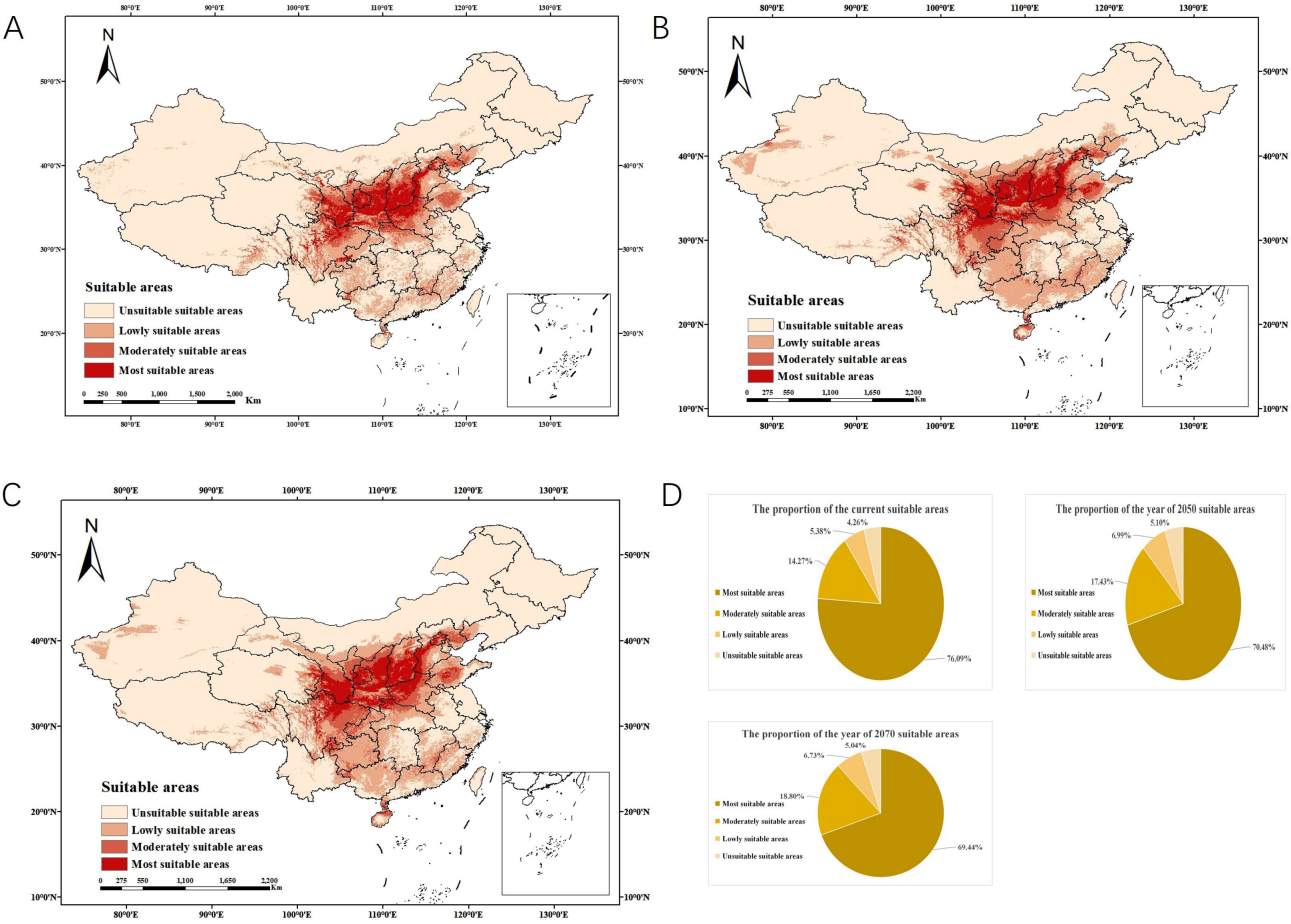
Distribution of suitable habitat area for *P. chinensis* across different time periods and their corresponding proportions. **(A)** Current prediction of *P. chinensis* habitat area. **(B)** Distribution of future habitat area of *P. chinensis* (2050). **(C)** Distribution of future habitat area of *P. chinensis* (2070). **(D)** Pie chart of the proportion of suitable areas from current to future.

The results showed that the area of the suitable areas, including highly suitable areas, moderately suitable areas, and areas with low suitability, was 2,295,360 km (Kato et al., 2019), accounting for 23.91% of China’s land area. Most suitable areas are mainly concentrated in central Shanxi Province, northern and western Henan Province, southern Shaanxi and Gansu provinces, southwestern Hebei Province, and northern Sichuan Province. The moderately suitable areas surround the highly suitable areas, primarily distributed in western Shandong, northern Anhui, central Shaanxi, northern Hunan, and other regions, forming a transitional zone from the core suitable areas to the periphery. The low suitability areas are even more widespread, as shown in **Figure 2A**.

### 3.5 Influence of future climate change on the distribution of *P. chinensis* areas

#### 3.5.1 Prediction results for future suitable areas for *P. chinensis* under the climate path of RCP4.5

Based on future climate data (2050, 2070) under the RCP4.5 pathway, the distributions of suitable areas of *P. chinensis* were predicted and the distribution map was drawn, as shown in **Figures 2B, C**. The suitable area for *P. chinensis* was calculated for different periods, and the distribution was influenced by environmental climate changes. The results showed that in 2050 and 2070, the suitable areas for sandflies will be 2,834,001 km² and 2,934,104 km², respectively, and the proportion of suitable areas will increase in 2050 and 2070. These results also show that the total suitable area for *P. chinensis* will increase in the future compared with current predictions (**Figure 2D**).

#### 3.5.2 Effects of climate change on the distribution of *P. chinensis* areas

**Figure 3:**
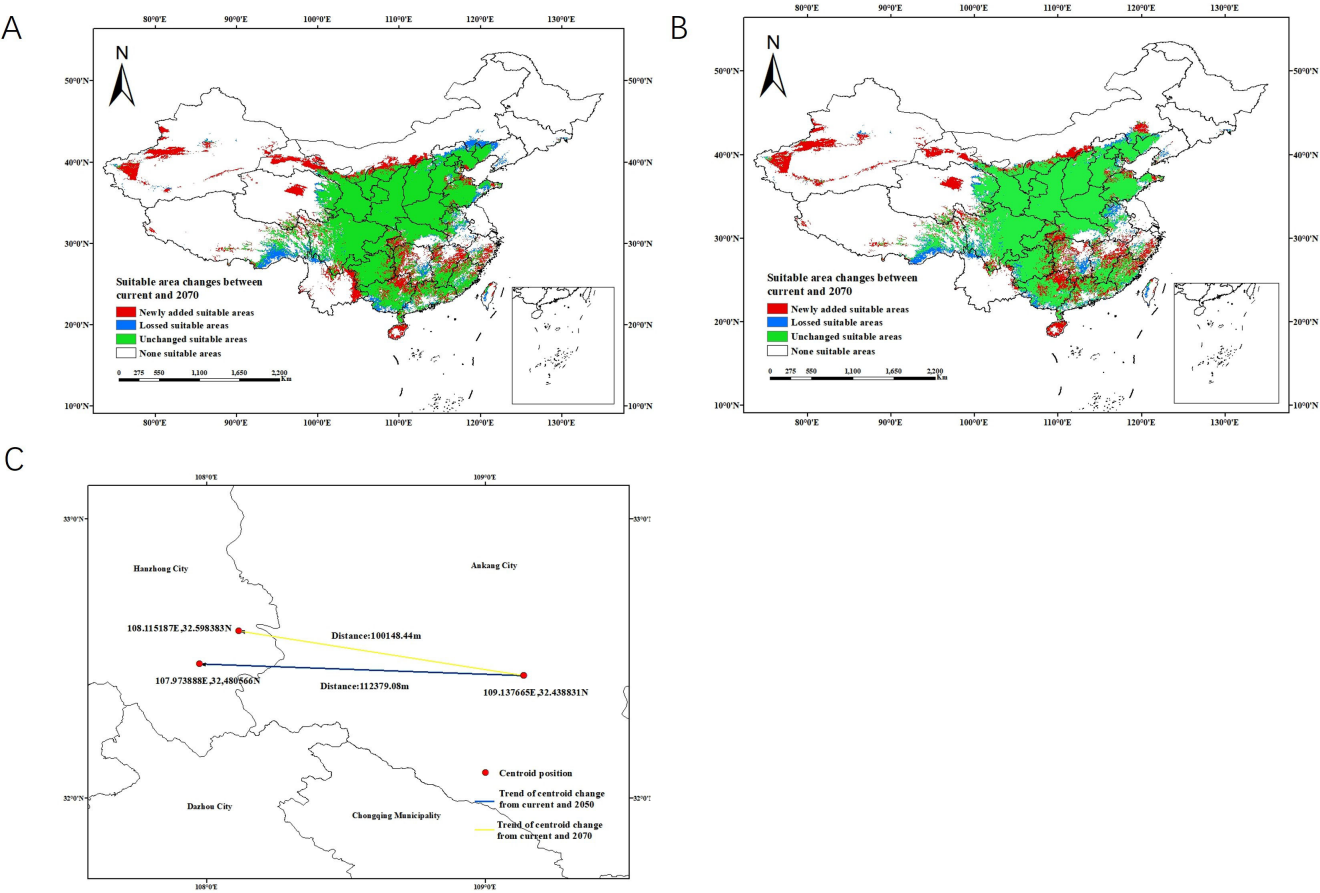
Changes in the distribution of suitable areas for *P. chinensis* from the present to 2050 and 2070, and the shift in the center of mass of suitable habitats under current and future scenarios. **(A)** Changes of the distribution of suitable areas (current to 2050). **(B)** Changes of the distribution of suitable areas (current to 2070). **(C)** Current and future (2050, 2070) center of mass transfer in the suitable area of *P. chinensis.*.

From the present to the future (2050, 2070), the suitable area pattern of *P. chinensis* in China exhibits significant spatial differentiation and dynamic evolution, with an overall expanding trend (**Figures 3A, B**). The newly added suitable areas are mainly concentrated in northwestern China (northern Xinjiang, the Hexi Corridor in Gansu, central and western Inner Mongolia), western Northeast China, and some mountainous areas in South China. This reflects that, under the background of climate warming, previously unsuitable high-latitude, high-altitude, and arid/semi-arid regions are gradually transforming into suitable habitats, showing a clear northward and westward expansion trend. In contrast, the lost suitable areas are sporadically distributed in eastern Northeast China, coastal South China, and parts of southwestern China, indicating that some low-latitude, low-altitude regions are losing suitability due to factors such as climate warming, resulting in local shrinkage of future suitable areas for *P. chinensis*. Overall, based on the stability of the core areas, the future suitable areas will present a spatial restructuring pattern characterized by “newly added areas in the northwest, and local losses in the northeast and south China.

#### 3.5.3 Effects of climate change on the center of mass in the suitable area for *P. chinensis*

The center of mass is a presumed point representing the concentrated central distribution of the suitable area simulation system. It has been calculated for different periods: the center of mass of the suitable area for *P. chinensis* is currently located in Dagui Town, Pingli County, Ankang City, Shaanxi Province (109.137665°E, 32.438831°N) at present. By 2050 and 2070, the centroids will shift northwestward by 112.37 km and 100.15 km, respectively. These points are located in Xiaoyang Town (32.480566°N, 107.973888°E), Zhenba County, Hanzhong City, Shaanxi Province, and Guanyin Town (32.598383°N, 108.115187°E), Zhenba County, Hanzhong City, Shaanxi Province, respectively (**Figure 3C**). The shift of the centroids is consistent with the differences between current and future *P. chinensis* areas. Notably, the newly suitable areas in northwestern China, including northern Xinjiang and central-western Inner Mongolia, satisfy the core environmental thresholds identified in this study: the minimum temperature of the coldest month in these regions is projected to rise from current values (often below -20 °C) to within the suitable range of -14.10 °C to -3.21 °C under climate change, and annual precipitation is projected to increase from <100 mm to closer to the 500–862 mm range, particularly in the Hexi Corridor and parts of Inner Mongolia. Thus, climate change will cause the suitable areas for *P. chinensis* to expand northwestward.

## 4 Discussion

In the 1950s, VL was one of the nine major parasitic diseases seriously prevalent in China (Hao et al., 2021). After prevention and control measures were implemented, it was successful controlled in the 1980s, and only sporadic cases remained in six provinces, namely Xinjiang, Inner Mongolia, Gansu, Sichuan, Shaanxi, and Shanxi (Luo et al., 2024).

However, although incidences of AVL and DT-ZVL have reduced with social development and ecological environment improvement, VL case numbers have grown, posing a serious threat to human life and health (Zhou et al., 2020). Therefore, it is very important to understand the distribution of *P. chinensis* in China. Eight provinces where VL is endemic were surveyed in this research. The results show that *P. chinensis* was mainly distributed in VL-endemic provinces; among them, the most samples of *P. chinensis* were collected in Linfen City, Jiexiu City, and Wuxiang Town in Shanxi Province. Guan et al. found that *P. chinensis* is prevalent across China, with the latitude of distribution ranging from 43°90′N, 125°50′E (northernmost), 23°40′N, 104°E (southernmost), 38°90′N, 100°40′E (westernmost), and 43°80′N, 126°60′E (easternmost) (Guan et al., 2016). Also, *P. chinensis* is the dominant species in Loess Plateau regions, mountains and plains (Zheng et al., 2024). In addition, VL is transmitted by sandflies, which are mainly found in Henan (Yang et al., 2023), Beijing (Jiang et al, 2021), Gansu (Han et al., 2021), Hebei (Khan et al., 2020), Shaanxi (Zhang et al., 2022), and Shanxi (Li et al., 2022). These results are consistent with the results of this survey. Moreover, this study found that *phlebotomus sichuanensis* and *sergentomyia squamirostris* are distributed in these areas.

The published literature has reported that the distribution of sandflies is influenced by the landscape (Guernaoui et al., 2006). In recent years, the primarily reason that the distributions of endemic and potential VL risk areas have increased across the world is global warming (Mohebali, 2013). To better understand the diffusion tendency of *P. chinensis*, it is important to study the impact of meteorological factor change on the geographic distribution of *P. chinensis*. Hence, the MaxEnt model was used to predict the distribution of *P. chinensis*.

As is known, the MaxEnt model can accurately predict the diffusion trends of suitable areas with a small number of points (Yun-Hai et al., 2016). However, the baseline data for this study were based on literature reviews and field surveys, which can make the MaxEnt model predictions more credible. Moreover, the size of the area under the ROC curve (AUC value) was used as a measure of model prediction accuracy. Moreover, the AUC value was proportional to the accuracy of the simulation results; the value of AUC in this simulation was 0.917 ± 0.007, illustrating excellent prediction for the distribution. The MaxEnt model, as an ecological niche model, can use environmental factor data to make accurate predictions (Matyukhina et al., 2014).

The results indicate that the greatest influence on the distribution of the habitat of sandflies was vegetation coverage (veg), following is the minimum temperature in the coldest month (bio_6), the means temperature of the driest quarter (bio_9) and the annual precipitation (bio_12) also had a greater influence on diffusion tendency.

The specific roles of suitable vegetation types in *P. chinensis* survival include: (1) temperate deciduous broad-leaved forests provide shaded, humid microenvironments under leaf litter and in soil crevices, which are essential for larval development and adult resting; (2) temperate semi-shrublands and short semi-shrubland deserts offer shelter from extreme temperatures and wind, as well as organic matter accumulation that supports breeding; (3) temperate mountain coniferous forests provide similar protective microhabitats at higher elevations.

Alten et al. found that the adult sandflies needed approximately 28 days to emerge at 32 °C, while they took about 245 days to emerge at 18 °C. However, at 15 °C, no specimen emerged; all environments had a relative humidity of 65 to 75% (Gao et al., 2017). Also, a sandfly temperature test was conducted: if the temperatures were above 40 °C with a relative humidity below 33% for 2 hours, all the sandflies died. Moreover, temperatures below 10 °C were harmful for sandfly larvae. Environmental temperature not only influences on sandfly growth and mortality, but also regulates the seasonal activity (Alten et al., 2003). Boussaa et al. reported that at temperatures of 11 °C to 36 °C, sandflies are more active in Morocco (Cross and Hyams, 1996). Moreover, the active period of sandflies is from June to September, which is closely related to temperature and humidity (Boussaa et al., 2005). When the temperature drops, usually after sunset, sandflies increases their activity (Faraj, 2011). In other words, the majority of sandflies become active and bite humans after sunset (Hanafi et al., 2007). It is recommended that people should take precautions against sandflies during this time, especially children.

Regarding the correlation between *P. chinensis* abundance and VL cases in Shanxi Province: While our study found that Shanxi had both the highest number of collected sandflies (particularly in Linfen, Jiexiu, and Wuxiang) and the highest number of VL cases reported in national surveillance data, caution is warranted in inferring a direct causal relationship. The abundance of *P. chinensis* is one of several factors contributing to VL transmission risk, along with reservoir host density, human exposure, and healthcare-seeking behavior. Further quantitative studies (e.g. correlation analysis between *P. chinensis* density and VL incidence at the county level) are needed to establish a direct correlation. Nevertheless, the spatial overlap suggests that Shanxi should be prioritized for VL surveillance and control.

From the results, we can see that the total area of suitable areas was 2,295,360 km (Kato et al., 2019), accounting for 23.91% of China’s land area. For 2050 and 2070, the figures are 29.52% and 39.56%, respectively. Both future predictions show expansion to the northwest, which is same for the center of mass transfer. This trend makes the prevention and control of kala-azar even more difficult. Therefore, we should be prepared to deal with the effects of climate change on the expansion of areas suitable for sandflies.

Several limitations of this study warrant discussion. First, as noted, we did not calculate sandfly population density, which may provide additional insights into transmission intensity. Future studies should incorporate density estimates. Second, our future projections were based solely on the RCP4.5 scenario; while this represents a moderate pathway consistent with Chinese policy, inclusion of RCP8.5 and RCP2.6 would provide a fuller range of possible outcomes. Third, the MaxEnt model relies on presence data only and does not account for species interactions (e.g., competition, predation) or dispersal limitations. Fourth, the 5 km buffer, while appropriate for matching climate data resolution, may not capture fine-scale microhabitat variations. And this study did not analyze the impact of human activities (e.g., land use change, population mobility, urbanization) on the distribution of *P. chinensis*. Future research should address these limitations by incorporating additional climate scenarios, density data, human activities and higher-resolution environmental layers.

## 5 Conclusions

This survey investigated the distribution of *P. chinensis* in the most endemic areas and areas with the greatest potential risk for VL in China; the most *P. chinensis* samples were collected in Shanxi Province. Meanwhile, the highest number of VL cases was also recorded in Shanxi Province. This may be positively correlated with the density of *P. chinensis*. The MaxEnt model determined that the distribution of *P. chinensis* will increase in a northwestward direction. More attention must be paid to central Shanxi Province, northern and western Henan Province, southern Shaanxi and Gansu provinces, southwestern Hebei Province, and northern Sichuan Province. Based on the findings, we propose the following targeted prevention and control strategies for these core suitable regions: (1) Establish sentinel surveillance sites in highly suitable areas (e.g., Linfen City, Jiexiu City, and Wuxiang Town in Shanxi) to surveillance the population dynamics of *P. chinensis* and VL case incidence in real time; (2) Implement integrated vector management measures, including indoor residual spraying with pyrethroid insecticides during peak *P. chinensis* activity months (June–September), and distribution of insecticide-treated bed nets in high-risk villages. Moreover, given the projected northwestward expansion of suitable areas under climate change, proactive surveillance should be extended to currently low-risk but emerging regions such as northern Xinjiang and central-western Inner Mongolia, where climate conditions are becoming increasingly suitable for *P. chinensis*.

## Data Availability

The original contributions presented in the study are included in the article. Further inquiries can be directed to the corresponding author.
